# Clinical Characteristics of Primary Hyperparathyroidism: 15-Year Experience of 457 Patients in a Single Center in China

**DOI:** 10.3389/fendo.2021.602221

**Published:** 2021-02-25

**Authors:** Xiaoyun Lin, Youben Fan, Zhenlin Zhang, Hua Yue

**Affiliations:** ^1^ Shanghai Clinical Research Center of Bone Diseases, Department of Osteoporosis and Bone Diseases, Shanghai Jiao Tong University Affiliated Sixth People’s Hospital, Shanghai, China; ^2^ Department of General Surgery, Thyroid and Parathyroid Center, Shanghai Jiao Tong University Affiliated Sixth People’s Hospital, Shanghai, China

**Keywords:** primary hyperparathyroidism, clinical features, follow-up, parathyroid carcinoma, bone pain

## Abstract

**Objective:**

Primary hyperparathyroidism (PHPT) is a common endocrine disorder of calcium metabolism. However, data concerning a large cohort of PHPT patients in the Chinese population are scarce. Thus, the objective of this study was to determine the general clinical signatures of 457 Chinese PHPT patients and explore the clinical characteristic differences between benign and malignant PHPT.

**Methods:**

A single-center retrospective study was designed. Medical records between preoperation and postoperative follow-up, were assessed and statistical analysis of the clinical data was performed.

**Results:**

Patients with PHPT aged 12–87 years, with a mean onset age of 56.16 ± 14.60 years, were included. Most patients (68.7%) in our center had symptomatic patterns described as bone pain (74.8%), urolithiasis (25.5%), fatigue (17.5%), and pathological fracture (13.1%), but an increasing tendency has been established in the proportion of patients with asymptomatic forms. Correlation analysis revealed that patients with higher serum levels of parathyroid hormone (PTH) and calcium presented higher serum levels of bone turnover markers (BTMs) and lower 25-hydroxy-vitamin D (25OHD) values (*P*<0.001). Gains in bone mineral density (BMD) at L1–4, the femoral neck and the total hip were observed 1–2 years after parathyroidectomy (9.6, 5.9, and 6.8%). Parathyroid carcinoma patients presented prominently higher serum PTH and calcium levels and BTMs and lower BMD at femoral neck and total hip than benign PHPT patients (*P*<0.05), while no significant differences in age, sex, and serum 25OHD concentration were observed between benign and malignant PHPT patients.

**Conclusions:**

PHPT should be paid attention to in the patients with bone pain. While, BMD and BTMs can differentiate parathyroid carcinoma from parathyroid adenoma and hyperplasia to some extent. In addition, anti-osteoporosis drugs could be used when necessary to avoid hip fractures in patients with parathyroid carcinoma.

## Introduction

Primary hyperparathyroidism (PHPT) is the most common cause of hypercalcemia, which has a female predominance (1, 2). Yeh *et al.* (3) conducted a study in a racially mixed population including 3.5 million enrollees with PHPT and found that the incidence was 92 and 46, 81 and 29, and 52 and 28 per 100,000 for females and males in Blacks, Whites, and Asians, respectively.

Since the advent of multichannel screening in Western countries in the 1970s, the epidemiology of PHPT has evolved from symptomatic profiles into asymptomatic profiles (4). Currently, the vast majority (>80%) of patients in the USA and Western Europe do not show symptoms and demonstrate a mild elevation in serum parathyroid hormone (PTH) and calcium (5). In contrast, reports from India, Thailand, and China in the past 5–10 years have concluded that most patients still manifest skeleton and kidney involvement as described in classical PHPT (6–8).

Parathyroid carcinoma is the rarest cause of PHPT, representing less than 1% of all PHPT, compared with approximately 80–85 and 15–20% of solitary parathyroid adenoma and parathyroid hyperplasia, respectively (9). The majority of parathyroid carcinoma cases are diagnosed postoperatively by histopathology on account of the difficulty of differentiating malignant PHPT from benign PHPT preoperatively. Considering that en bloc resection is the gold standard treatment for parathyroid carcinoma and that the identification of parathyroid carcinoma prior to surgery can provide options for surgical procedures (10), we conducted a study to compare benign and malignant PHPT.

To the best of our knowledge, to date, there are no studies available on the clinical and biochemical traits of PHPT in more than 400 Chinese patients (8, 11–15). Our center has reported 260 patients with PHPT from 2005 to 2016; regrettably, the study lacks bone turnover markers (BTMs), bone mineral density (BMD), and postoperative follow-up data. Meanwhile, with the popularity of routine biochemical screening in China (4), an increasing number of patients with PHPT have been recognized in our center in recent years. Therefore, herein, we expanded all cases, gathered more detailed data, and improved the defects mentioned above in an attempt to elaborate the clinical and biochemical features of Chinese patients with PHPT in 2005–2019.

## Subjects and Methods

### Subjects

The study was approved by the Ethics Committee of Shanghai Jiao Tong University Affiliated Sixth People’s Hospital. Written informed consent was obtained from all participants. A total of 457 subjects were enrolled in this study from January 1, 2005, to July 30, 2019, including one multiple endocrine neoplasm one case and one multiple endocrine neoplasm 2A case confirmed genetically. Symptomatic PHPT patients were diagnosed based on concomitant symptoms (bone pain, nephrolithiasis, pathological fractures, polydipsia and polyuria, digestive symptoms, neuropsychiatric manifestations, etc.), persistent hypercalcemia, and serum PTH excess. Asymptomatic PHPT was defined as the absence of symptoms and the presence of hypercalcemia and elevated or inappropriately normal PTH concentrations (9).

Patients with secondary (e.g., vitamin D deficiency, kidney insufficiency, intestinal calcium malabsorption) or tertiary hyperparathyroidism (hyperphosphatemia, renal transplantation) were excluded.

The age of onset was recorded according to the first identification of symptoms related to PHPT for symptomatic patients or an elevation in serum calcium or PTH concentrations for asymptomatic patients. Surgery was performed when patients met the criteria (16). The tumor specimens were diagnosed by professional pathologists, and the largest single diameter from the pathology report was utilized as the tumor size (17). The definite histological criteria for the diagnosis of parathyroid carcinoma were as follows (18): i) sheets or lobules of tumor cells with interspersed fibrous bands; ii) extremely abundant mitosis; iii) necrosis of tumor tissues; iv) capsular invasion; and v) vascular invasion. Follow-up data were recorded when available. The persistence of PHPT was defined as hypercalcemia within 6 months of parathyroidectomy, and the recurrence of PHPT was defined as hypercalcemia presenting after a normocalcemic interval at more than 6 months after parathyroidectomy (16).

### Clinical Features

The basic information was collected, including sex, age at the time of diagnosis, and body mass index (BMI). Radiographs of skeletons were performed to assess bone involvement. Renal ultrasound was carried out to evaluate nephrolithiasis. Parathyroid ultrasound and/or ^99^mTc-sestamibi scintigraphy were conducted preoperatively to assist in locating the abnormal tissues.

### Laboratory Tests

Relevant biochemical tests included intact serum PTH, serum 25-hydroxy-vitamin D (25OHD), serum calcium, phosphate, β-crosslaps of type 1 collagen containing cross-linked C-telopeptide (β-CTX), serum osteocalcin (OC) in the form of an N-terminal mid-molecule fragment, total alkaline phosphatase (ALP), creatinine, albumin, hemoglobin, triglycerides (TG), total cholesterol (TC), high-density lipoprotein cholesterol (HDL-C), low-density lipoprotein cholesterol (LDL-C), and fasting plasma glucose (FPG). The serum calcium levels were adjusted by the following formula when there was any aberration in albumin: (40—serum albumin concentration in grams per liter)×0.2+ measured total serum calcium in millimole per liter.

Serum PTH, 25OHD, β-CTX, and OC concentrations were measured using an automated Roche electrochemiluminescence system. The serum calcium, phosphate, and ALP levels were measured using a Hitachi 7600-020 automatic biochemistry analyzer.

### BMD Measurements

The BMD (g/cm^2^) of the lumbar spine (L1–4), left femoral neck, and total hip was measured using dual-energy X-ray absorptiometry (DXA). Validated DXA scans were available for 180 patients assessed by Lunar software (Lunar, Madison, WI, USA). T- and Z-scores were obtained using the reference norms of the manufacturer. The T-score was used for postmenopausal women and men aged 50 and older, and the Z-score was used for premenopausal women and men under 50. The Lunar devices were calibrated daily, and the measurements were conducted by the same well-trained technologist.

### Statistical Analyses

All data were analyzed using IBM SPSS Statistics (version 26.0; SPSS Inc., Chicago, Illinois). The normally distributed variables are presented as the mean ± SD, and between-group differences were assessed with independent-samples *t* test or one-way ANOVA as appropriate. The medians (25th and 75th percentiles) are presented for non-normally distributed variables, and the Mann-Whitney *U* test or Kruskal-Wallis test were applied to compare intergroup differences. Categorical variables are described as frequencies or percentages, and intergroup comparisons were analyzed with the χ^2^ test or Fisher’s exact probability test. Correlations between variables were analyzed with the Spearman rank correlation coefficient. A two-tailed value of *P*<0.05 was considered statistically significant.

## Results

### Demographics and General Characteristics of Patients with PHPT

The study group included 457 patients: 352 (77.0%) were females, 105 (23.0%) were males (female-to-male ratio of 3.4:1), 314 (68.7%) were symptomatic, and 143 (31.3%) were asymptomatic, with a mean age and onset age of 58.44 ± 14.11 and 56.16 ± 14.60 years, respectively.

**Figure 1:**
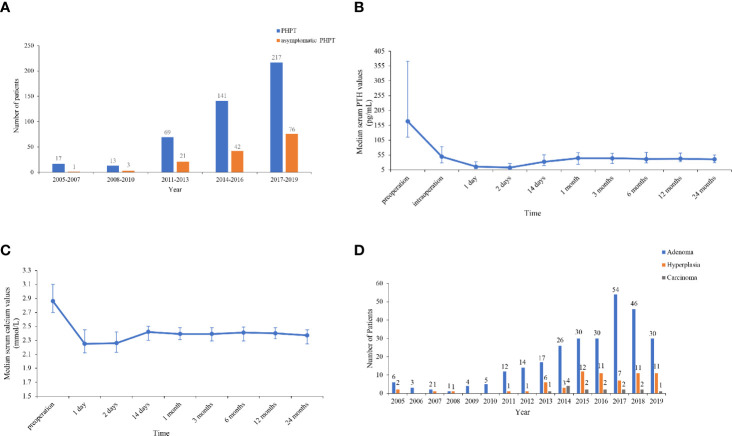
**(A)** Number of total and asymptomatic patients in 2005–2019. The number of patients with total primary hyperparathyroidism (PHPT) markedly increased by 11.8 times from 2005–2007 to 2017–2019, and the proportion of asymptomatic patients increased by 5 times from 2005–2007 to 2017–2019. **(B, C)** Trends of serum parathyroid hormone (PTH) and calcium levels pre- and intraoperatively and within 24 months after parathyroidectomy. Serum PTH and calcium levels decreased to the lower limit of normal 1–2 days after resection, then increased to a peak of normal range at 1 month and then reached a rather sustainable plateau at 6–24 months. **(D)** Number of patients with parathyroid adenoma, hyperplasia, and carcinoma in 2005–2019. The number and proportion of patients with parathyroid adenoma, hyperplasia and carcinoma were 280 (77.6%), 67 (18.6%), and 14 (3.9%), respectively.

The numbers of patients with PHPT and asymptomatic PHPT have both increased over the past 15 years. The number of patients with total PHPT markedly increased by 11.8 times from 2005–2007 to 2017–2019, and the number of patients diagnosed in 2017–2019 accounted for 47.5% of all cases in 2005–2019. Furthermore, the proportion of asymptomatic patients increased by five times from 5.9 (1/17) to 35.0% (76/217) from 2005–2007 to 2017–2019 (**Figure 1A**).

A total of 361 patients with PHPT who underwent parathyroidectomy underwent parathyroid ultrasound or ^99m^Tc-sestamibi scintigraphy preoperatively to localize the parathyroid lesions.

There were 335 patients examined by parathyroid ultrasound, and 86.6% (290/335) were true positive. Among 290 patients, 221 had adenoma, 59 had hyperplasia, and 10 had carcinoma, based on the results of ultrasonic diagnosis. In addition, 46.6% (135/290) of the patients’ ultrasonic diagnosis corresponded to the final histopathology. Among 135 patients, 126 had adenoma, 3 had hyperplasia, and 6 had carcinoma.

There were 303 patients examined by ^99m^Tc-sestamibi scintigraphy, and 95.4% (289/303) were true positive. Among these 289 patients, 224 had adenoma, 60 had hyperplasia, and 5 had carcinoma, according to the diagnosis by sestamibi, and 44.3% (128/289) of the patients’ diagnosis by sestamibi were complied with the pathological results. Among 128 patients, 125 had adenoma, none had hyperplasia, and 3 had carcinoma.

A significant difference was observed between the two imaging techniques in assisting with the localization of tumors (*P<*0.001).

### Clinical and Biochemical Features of PHPT

The clinical and biochemical characteristics of patients with PHPT are enumerated in **Tables 1**–**3**. The most frequent manifestations of symptomatic patients were bone pain (74.8%), urolithiasis (25.5%), fatigue (17.5%), pathological fracture (13.1%), polydipsia and polyuria (10.8%), and neuropsychiatric disorders (7.0%). A total of 85/160 (53.1%) patients who were postmenopausal women or men aged 50 and older had BMD T-scores ≤ −2.5 at any site, with the following specific site involvement: 56 (65.9%) in L1–4, 36 (42.4%) in the femoral neck, and 30 (35.3%) in the total hip. Meanwhile, 9/20 (45.0%) patients who were premenopausal women or men under 50 had BMD Z-scores ≤−2.0 at one or more sites, with the following specific site involvement: 9 (100.0%) in L1–4, 6 (66.7%) in the femoral neck, and 8 (88.9%) in the total hip.

**Table 1:** Clinical Spectrum of Symptomatic patients with PHPT (n=314).

Symptoms	No. (%)
Bone pain	235 (74.8%)
Urolithiasis	80 (25.5%)
Fatigue	55 (17.5%)
Pathological fracture	41 (13.1%)
Polydipsia and polyuria	34 (10.8%)
Neuropsychiatric disorders	22 (7.0%)
Palpitation and chest distress	16 (5.1%)
Nausea and vomiting	14 (4.5%)
Constipation	4 (1.3%)
Pruritus	2 (0.6%)

**Table 2:** Clinical and biochemical characteristics of total primary hyperparathyroidism (PHPT), parathyroid adenoma, hyperplasia, and carcinoma cases.

	Total PHPT	Adenoma	Hyperplasia	Carcinoma	*P*-value
No.	457	280	67	14	
Age, y	58.44 ± 14.11^*^	58.24 ± 14.02	56.10 ± 13.18	54.07 ± 12.09	0.320
Age<50 years	21.4%	21.4%	28.4%	50.0%^c^	**0.033**
Age of onset, year	56.16 ± 14.60	56.31 ± 14.48	54.48 ± 13.60	54.17 ± 12.41	0.585
Sex (female: male)	3.4:1	3.1:1	5.7:1	2.5:1	0.182
BMI, kg/m^2^	23.65 ± 3.34	23.59 ± 3.25	23.78 ± 3.17	20.92 ± 3.47^c^ ^,^ ^d^	**0.033**
Tumor size, cm	1.80 (1.30–2.50)	1.80 (1.50–2.50)^d^	1.15 (1.00–2.00)	3.75 (3.00–4.88)^b^ ^,^ ^c^	**<0.001**
Serum PTH, pg/ml	168.35 (115.30–370.98)^†^	200.50 (128.28–431.50)^d^	152.50 (103.20–285.65)	1,315.00 (530.13–2,060.25)^a^ ^,^ ^b^	**<0.001**
Serum intra-PTH, pg/ml	49.42 (28.64–83.05)	47.60 (28.16–83.06)	44.25 (28.09–60.86)	171.30 (80.96–362)^a^ ^,^ ^b^	**0.005**
Serum post-PTH, pg/ml	15.61 (8.76–31.11)	15.26 (7.90–29.37)	20.53 (11.82–33.55)	18.98 (10.42–76.90)	0.067
Albumin-corrected serum calcium, mmol/L	2.83 (2.70–3.10)	2.86 (2.72–3.11)	2.79 (2.66–3.03)	3.30 (3.13–4.03)^a^ ^,^ ^b^	**<0.001**
Hypercalcemic crisis (>3.5 mmol/L)	8.3%	7.3%	7.0%	38.5%^a^ ^,^ ^b^	**0.005**
Serum phosphate, mmol/L	0.83 ± 0.18	0.81 ± 0.23	0.86 ± 0.16	0.84 ± 0.38	0.408
Serum 25 (OH)D, ng/ml	14.03 (10.12–17.74)	13.00 (10.01–16.62)	15.90 (8.63–20.64)	11.05 (8.83–14.79)	0.193
Serum β-CTX, ng/L	863.30 (453.25–1,321.75)	1,007.30 (647.33–1,536.50)^b^	563.00 (266.35–772.18)	2,972.00 (2,457.50–5,283.50)^b^ ^,^ ^c^	**<0.001**
Serum OC, ng/ml	41.72 (24.98–71.21)	49.57 (31.39–97.71)^d^	31.91 (22.10–50.94)	300.00 (199.60–300.00)^a^ ^,^ ^b^	**<0.001**
Serum ALP, U/L	104 (76–150)	114 (83–161)	86 (70–124)	434 (155–769)^a^ ^,^ ^b^	**<0.001**
Serum creatinine, μmol/L	59 (51–76)	60 (51–75)	56 (49–72)	81 (52–129)	0.159
Hemoglobin, g/L	128.41 ± 17.73	128.32 ± 19.56	128.57 ± 11.25	120.93 ± 28.36	0.894
Albumin, g/L	44.51 ± 4.13	45.07 ± 6.76	44.40 ± 4.37	43.29 ± 6.29	0.473
TG, mmol/L	1.47 (0.90–1.99)	1.59 (1.04–2.14)	1.33 (0.78–1.89)	1.67 (0.93–2.41)	0.782
TC, mmol/L	4.75 ± 1.05	4.70 (4.11–5.45)	4.74 (3.55–5.14)	4.65 (3.87–5.67)	0.853
HDL-C, mmol/L	1.25 ± 0.36	1.18 ± 0.30	1.38 ± 0.49	1.55 ± 0.66	0.318
LDL-C, mmol/L	2.78 ± 0.83	2.83 ± 0.93	2.58 ± 0.79	2.38 ± 0.33	0.463
FBG, mmol/L	5.32 (4.92–5.98)	5.32 (4.92–5.87)	5.24 (4.81–6.03)	5.21 (4.75–5.65)	0.632

^*^Normally distributed data are shown as the mean ± SD.

^†^Non-normally distributed data are shown as the median (interquartile range).

25OHD, 25-hydroxy-vitamin D; β-CTX, β-crosslaps of type 1 collagen containing cross-linked C-telopeptide; OC, serum osteocalcin in the form of N-terminal mid-molecule fragment, ALP, total alkaline phosphatase; TG, triglycerides; TC, total cholesterol; HDL-C, high-density lipoprotein cholesterol; LDL-C, low-density lipoprotein cholesterol; FPG: fasting plasma glucose.

Normal range: PTH: 15–65 pg/ml, calcium: 2.08–2.60 mmol/L, phosphate: 0.80–1.60 mmol/L, 25OHD: >30 ng/ml, β-CTX: 278–540 ng/L(19), OC: 13.07–27.68 ng/ml(19), ALP: 15–112 U/L, creatinine: 53.0–115.0 mol/L, hemoglobin: 130–175 g/L, albumin: 35–55 g/L, TG: 0.45–1.81 mmol/L, TC: 2.80–5.90 mmol/L, HDL-C: 0.90–1.68 mmol/L, LDL-C: 2.84–4.10 mmol/L, FPG: 3.90–5.80 mmol/L.

a P < 0.01 compared to parathyroid adenoma.

b P < 0.01 compared to parathyroid hyperplasia.

c P < 0.05 compared to parathyroid adenoma.

d P < 0.05 compared to parathyroid hyperplasia.

Bold numbers represent P < 0.05.

**Table 3:** Bone mineral density (BMD) of total primary hyperparathyroidism (PHPT), parathyroid adenoma, hyperplasia, and carcinoma cases.

	Total PHPT	Adenoma	Hyperplasia	Carcinoma	*P*-value
L1–4					
g/cm^2^	0.88 ± 0.17	0.84 ± 0.13	0.89 ± 0.13	0.87 ± 0.24	0.388
T-scores	−1.98 ± 1.42	−2.30 ± 1.07	−1.80 ± 1.13	−1.95 ± 1.98	0.287
Z-scores	−0.68 ± 1.40	−0.90 ± 1.09	−0.97 ± 1.15	−0.66 ± 2.28	0.914
Femoral neck					
g/cm^2^	0.72 ± 0.12	0.70 ± 0.*10* ^a^	0.79 ± 0.11	0.64 ± 0.09^b^	**0.004**
T-scores	−1.71 ± 0.92	−1.90 ± 0.80^a^	−1.08 ± 0.91	−2.40 ± 0.90^b^	**0.002**
Z-scores	−0.41 ± 0.91	−0.54 ± 0.88	−0.07 ± 0.81	−1.01 ± 1.50	0.118
Total hip					
g/cm^2^	0.76 ± 0.12	0.74 ± 0.11^b^	0.83 ± 0.13	0.61 ± 0.07^b^	**0.004**
T-scores	−1.61 ± 0.98	−1.80 ± 0.*87* ^b^	−1.06 ± 1.02	−2.78 ± 0.61^a^	**0.003**
Z-scores	−0.62 ± 0.96	−0.77 ± 0.87	−0.33 ± 0.95	−1.78 ± 1.29^b^	**0.032**

Values are presented as mean ± SD.

a P < 0.01 compared to parathyroid hyperplasia.

b P < 0.05 compared to parathyroid hyperplasia.

The median β-CTX and OC values were 863.30 ng/L (interquartile range: 453.25–1,321.75 ng/L) and 41.72 ng/ml (interquartile range: 24.98–71.21 ng/ml) in the cohort, which were above the established reference range of healthy Chinese subjects (19). Furthermore, 43.7% (190/434) of patients had elevated ALP values above the upper limit of normal, which suggested active bone turnover in these patients. Additionally, vitamin D deficiency (<20 ng/ml) was found in 83.0% (234/282) of patients with PHPT.

Correlation analysis revealed that patients with higher serum PTH and calcium levels had higher serum levels of β-CTX (*r*=0.665, *P*<0.001), OC (*r*=0.605, *P*<0.001) and ALP (*r*=0.675, *P*<0.001), and larger tumor sizes (*r*=0.579, *P*<0.001) but exhibited lower serum 25OHD concentrations and younger age (*P*<0.001) (**Table 4**).

**Table 4:** Correlation analyses of serum parathyroid hormone (PTH) and calcium levels and other clinical and biochemical parameters.

	Serum PTH		Serum calcium	
	*r*	*P* value	*r*	*P* value
Age	−0.316	<0.001	−0.273	<0.001
β-CTX	0.665	<0.001	0.582	<0.001
OC	0.605	<0.001	0.416	<0.001
25OHD	−0.211	<0.001	−0.111	<0.001
ALP	0.675	<0.001	0.386	<0.001
Serum creatinine	0.345	<0.001	0.294	<0.001
Hemoglobin	−0.277	<0.001	−0.292	<0.001
Tumor size	0.579	<0.001	0.489	<0.001

### Postoperative Follow-Up Findings

Parathyroid surgery was performed on 361 patients, and follow-up data were available for 228 patients, with a median follow-up duration of 6.5 months (range, 0.5–120 months). The trends of serum PTH and calcium levels within 24 months after surgery are listed in **Figures 1B, C**. Serum PTH and calcium levels dramatically decreased to the lower limit of normal 1–2 days after resection, then increased to a peak of normal range at 1 month and subsequently reached a rather sustainable plateau at 6–24 months. Moreover, 12–24 months after parathyroidectomy, patients had BMD gains of 9.6, 5.9, and 6.8% on average at L1–4, the left femoral neck and the total hip, respectively.

Persistence was found in one (0.4%) patient who was confirmed to have an ectopic adenoma in a second surgery after a follow-up of 6 months, and recurrence was noted in six (2.6%) patients at a median duration of 42.0 months (range, 12–108 months). Among them, four had parathyroid carcinoma recurring at a median follow-up of 36 months (two underwent parathyroidectomy twice, and the others underwent parathyroidectomy three times), and two developed a second adenoma in a previously normal gland after the initial surgery.

### Comparison of Patients with Parathyroid Adenoma, Hyperplasia, and Carcinoma

Of 361 patients who were treated with surgery, 76.8% (276/361) had a single parathyroid adenoma, and 1.1% (4/361) had multiple adenomas. A total of 18.6% (67/361) of the patients had parathyroid hyperplasia, four of which had multigland hyperplasia. A total of 3.9% (14/361) had parathyroid carcinoma. Strikingly, the incidence of parathyroid carcinoma increased from 0 to 4.2% in the period of 2005–2013 but substantially decreased by 80.0% from 12.1 to 2.4% over the past 5 years (**Figure 1D**).

In seven patients, the parathyroid glands were ectopic: three were in the superior mediastinum, one was in the substernal region, one was in the sternoclavicular joint, one was beside the common carotid artery and innominate artery, and one was beside the trachea.

Compared with parathyroid adenoma and hyperplasia, carcinoma cases presented higher serum PTH, calcium, and ALP levels (*P*<0.001) and larger tumor sizes (*P*<0.001). Moreover, serum β-CTX and OC values were also higher in carcinoma cases than in adenoma and hyperplasia cases (*P*<0.001); however, no difference was noted in BMD at all regional sites except femoral neck and total hip between benign and malignant PHPT (**Table 3**). Similarly, no differences were observed among patients with parathyroid adenoma, hyperplasia, and carcinoma in terms of sex, age, onset age, or serum post-PTH, serum 25OHD, creatinine, hemoglobin, albumin, TG, TC, HDL-C, LDL-C, and FBG levels (**Table 2**).

## Discussion

Our retrospective study of 457 patients with PHPT showed increasing trends of both symptomatic and asymptomatic patients in a single center in China during a 15‐year study period (2005–2019). As far as we know, this is the largest cohort to be reported in China so far (8, 11–13, 15, 20). The number of diagnosed and treated PHPT cases in 2017–2019 was practically half of all the cases over 15 years. Reasons for the tremendous increase may lie in the enhanced consciousness of osteoporosis assessment, bringing about the increased measurement of serum PTH and calcium and the wider application of routine biochemical screening in China at present. Unlike the USA and Western Europe, whose PHPT patients were primarily asymptomatic (>80%) (21), our center had a proportion of asymptomatic patients of only 31.3%, but the proportion of asymptomatic patients continued to increase over the past 15 years. Although there might be a possibility that we omitted certain mild symptoms of patients with PHPT when collecting medical histories clinically and mistakenly classified these patients as asymptomatic, given that the determination of serum calcium became increasingly common in nearly a decade at our center, we can still draw the conclusion that the proportion of our asymptomatic patients exhibited an increasing tendency.

In Western countries, less than 5% of PHPT patients suffer from overt bone disease (22, 23). Conversely, at our center, skeleton involvement was the most predominant symptom, with a proportion of 74.8%, which is also higher than that reported previously in Chinese PHPT patients. Since our institute is specialized in the diagnosis and treatment of bone diseases, most patients came to seek medical consultation for bone pain and ultimately diagnosed as PHPT. Such patients usually have longer durations of disease and varying degrees of bone involvement. Therefore, we suggest that for patients with bone disease, PHPT should be screened. On the other hand, due to the characteristic of our center, the high proportion of bone pain in PHPT patients in our study may not be widely representative in the population. There might exist an overestimation of the proportion of patients with bone pain, thus further multi-center studies should be carried out.

PHPT is usually accompanied by insufficient (20–29 ng/ml) or deficient (<20 ng/ml) serum 25OHD levels (24). Preceding studies have reported 93% of 73 PHPT patients in French and 81% of 289 PHPT patients in Northern Europe had vitamin D deficiency (25, 26). Similarly, our study revealed that more than 80% of patients suffered from 25OHD deficiency. On the other hand, our study indicated there was no difference in vitamin D levels between benign and malignant PHPT. Nevertheless, since vitamin D is associated with a series of confounding factors such as sunlight exposure, dietary supplements and lifestyles, etc (27), the explanation of vitamin D is relatively complex. It has been reported that lower 25OHD leads to higher bone turnover and lower BMD values (28). Accordingly, international guidelines suggested that vitamin D should be supplemented in PHPT patients to reach a serum level of 25OHD>20 ng/ml, even up to 30 ng/ml (29). It is worth mentioning that guidelines also advised patients with PHPT not to restrict dietary calcium intake. Instead, 1,000–1,200 mg per day of dietary calcium supplementation will minimize bone loss (21).

PHPT is an important disease that causes secondary osteoporosis. In our study, the BMD at L1–L4, the left femoral neck and the total hip were decreased in patients with PHPT, with half of the patients having osteoporosis. Meanwhile, patients with parathyroid carcinoma had lower BMD at the femoral neck and the total hip than that at L1–L4, in accordance with the finding that patients with PHPT tended to have a more severe reduction in cortical bone as assessed by DXA (23). On the other hand, many studies have reported an improvement in BMD after curative parathyroidectomy. A study by Sitges-Serra *et al*. reported BMD increases of 1.3, 0.4, and 2.3% for the femoral neck, total hip, and lumbar spine sites, respectively, 1 year after surgery (30). An observational study by Rubin *et al.* (31) concluded that BMD increased by 12 and 10% at the lumbar spine and femoral neck, respectively, 10 years after successful surgery. Moreover, recent studies found that PHPT patients with a typical clinical profile may receive more robust skeletal benefits from parathyroidectomy than patients with the mild type (32). Thus, the high rate of BMD improvement in patients with PHPT in our center may be explained by the fact that bone involvement was most commonly seen in those patients; however, the BMD at hip is lower in patients with parathyroid carcinoma, and hip fracture is the most serious osteoporotic fracture, related with high mortality for 28% at 6 months and 33% at 1 year (33), thus, patients with parathyroid carcinoma should be alert to the occurrence of hip fracture, and active anti-osteoporosis treatment could be provided after parathyroidectomy if necessary.

The diagnosis of parathyroid carcinoma preoperatively is difficult due to a definite symptomatology overlap between benign and malignant PHPT. Reportedly, patients with malignant PHPT presented serum PTH concentrations 5–10 times the upper limit of normal (18) compared to those with benign PHPT, who had serum PTH values within 2 times above the reference range (5). Likewise, hypercalcemic crisis (>3.5 mmol/L) is usually found in up to 10% of patients with parathyroid carcinoma (34), as opposed to patients with parathyroid adenoma or hyperplasia, whose serum calcium levels are generally at most 0.25 mmol/L above the upper limit of normal (5). In our study, PTH values were markedly higher than 10 times the normal range, and hypercalcemic crisis accounted for 38.5% of the carcinoma cases. Moreover, we noticed that in our study, the BTMs of parathyroid carcinoma were significantly higher than those of either parathyroid adenoma or parathyroid hyperplasia, indicating that patients with malignant PHPT had much more serious bone involvement than those with benign forms of PHPT. Hence, the BTMs could be used for differentiating benign and malignant PHPT to some extent.

Patients with parathyroid carcinoma have a recurrence rate of up to 50%, and recurrence occurs more often 2–3 years after parathyroidectomy (35). Studies have reported that incomplete tumor removal, age>65 years, preoperative calcium levels>3.75 mmol/L and tumor size>4 cm are negative factors of parathyroid carcinoma prognosis (10, 36). The recurrence rate of patients who undergo complete resection of the tumor at initial surgery will decrease to 33% (18). Therefore, it is of great necessity to recognize parathyroid carcinoma before surgery. Patients with parathyroid carcinoma manifest symptoms similar to those of typical PHPT patients; hence, biochemical markers are essential signs of suspicion of carcinoma. Several studies have focused on predicting parathyroid carcinoma preoperatively and concluded that the serum ALP level has the best capability to predict parathyroid carcinoma based on the AUC value in the ROC curve, followed by the serum PTH level; serum calcium had the lowest value of all three parameters (37–39). In this study, five of the 14 patients with parathyroid carcinoma have undergone en bloc resection at initial surgery, and no recurrence was observed during the period of follow-up. These five patients manifested high levels of serum PTH, calcium, and BTMs, and were suspected of carcinoma by ultrasound or sestamibi preoperatively. During intraoperative exploration, the tumors were relatively large and adhered to the adjacent tissues, therefore the en bloc resection was conducted.

There were some strengths of our study. First, the sample size was large enough to ensure a more precise description of patients with PHPT in China. Second, follow-up data were obtained to better grasp the surgical outcomes and long-term development of PHPT. However, certain limitations should be mentioned. Despite the large cohort, it is a single-center study, and the patients included principally came from Shanghai and neighboring provinces. Consequently, the result cannot be extrapolated to the general Chinese population, so multiple-center studies are needed to provide a comprehensive characterization of the disease in China. Furthermore, there was selection bias because our hospital is a tertiary referral unit, and patients from other provinces presented more severe symptoms. On the other hand, to reduce the selection bias, we ensured a certain sample size in the research and gathered as much clinical and biochemical data as possible.

In conclusion, our monocentric, retrospective study revealed that patients with PHPT at our center still exhibited a symptomatic pattern. Because of the heterogeneity of the clinical manifestations of the PHPT patients in our cohort, screening serum calcium in patients presenting with bone pain, urolithiasis, or neuropsychiatric disorders must be seriously considered. In addition, parathyroid carcinoma is characterized by more severe clinical traits and a higher recurrence rate than its benign counterparts, so follow-up should be strengthened for patients with parathyroid carcinoma after parathyroidectomy.

## Data Availability Statement

The raw data supporting the conclusions of this article will be made available by the authors, without undue reservation.

## Ethics Statement

The studies involving human participants were reviewed and approved by Ethics Committee of Shanghai Jiao Tong University Affiliated Sixth People’s Hospital. Written informed consent to participate in this study was provided by the participants’ legal guardian/next of kin.

## Author Contributions

XL and YF conducted the study and analyzed the data. YF contributed to Parathyroidectomy and manuscript preparation. XL wrote the draft manuscript. ZZ and HY supervised the study and revised the manuscript. All authors contributed to the article and approved the submitted version.

## Funding

This work was supported by the National Key Research and Development Program of China (No. 2018YFA0800801); National Natural Science Foundation of China (NSFC) (Nos. 81974126; 81770874 and 81670718); the Clinical Science and Technology Innovation Project of Shanghai Shenkang Hospital Development Center (No. SHDC12018120); Shanghai Key Clinical Center for Metabolic Disease, Shanghai Health Commission Grant (No. 2017ZZ01013).

## Conflict of Interest

The authors declare that the research was conducted in the absence of any commercial or financial relationships that could be construed as a potential conflict of interest.
